# Prospective Associations between Dietary Patterns and Abdominal Obesity in Middle-Aged and Older Korean Adults

**DOI:** 10.3390/foods12112148

**Published:** 2023-05-26

**Authors:** Kyung Won Lee, Min-Sook Kang, Seung Jae Lee, Haeng-Ran Kim, Kyeong-A Jang, Dayeon Shin

**Affiliations:** 1 Department of Home Economics Education, Korea National University of Education, Cheongju 28173, Republic of Korea; 2 Department of Agro-Food Resources, National Institute of Agricultural Sciences, Rural Development Administration, Wanju 55365, Republic of Korea; 3 Department of Nutritional Science and Food Management, Ewha Womans University, Seoul 03760, Republic of Korea; 4 Department of Food and Nutrition, Inha University, Incheon 22212, Republic of Korea

**Keywords:** dietary pattern, factor analysis, abdominal obesity, KoGES, Korean adults

## Abstract

This study aimed to identify major dietary patterns associated with abdominal obesity in middle-aged and older Korean adults. Data from the Korean Genome and Epidemiology Study were used. A total of 48,037 Korean adults aged ≥40 years without abdominal obesity at baseline were followed-up. Dietary assessment was conducted using a validated 106-item food-frequency questionnaire, and dietary patterns were identified using factor analysis. Abdominal obesity was defined as a waist circumference of ≥90 cm for men and ≥85 cm for women, according to the Korean Society for the Study of Obesity. Multivariable Cox proportional-hazards models were used to calculate the hazard ratios (HRs) and 95% confidence intervals (CIs) for the future risk of abdominal obesity for each dietary pattern after adjusting for potential covariates. After an average follow-up of 4.89 years, we reported 5878 cases (1932 men and 3946 women) of abdominal obesity. Based on factor analysis, three major dietary patterns were identified in both men and women: the “healthy”, “coffee and sweets”, and “multi-grain” patterns. In the fully adjusted model, the “healthy” pattern was inversely associated with the incidence of abdominal obesity (HR for fourth vs. first quartile: 0.86; 95% CI: 0.75–0.98; *p* for trend = 0.0358 for men; HR for fourth vs. first quartile: 0.90; 95% CI: 0.83–0.99; *p* for trend = 0.0188 for women), whereas the “coffee and sweets” pattern was positively associated with it (HR for fourth vs. first quartile: 1.23; 95% CI: 1.08–1.40; *p* for trend = 0.0495 for men; HR for fourth vs. first quartile: 1.14; 95% CI: 1.04–1.25; *p* for trend = 0.0096 for women). In contrast, the “multi-grain” pattern in men and women showed no significant association with the incidence of abdominal obesity. Diets rich in colorful vegetables, seaweeds, mushrooms, tubers, fruits, soy products, and fish and low in coffee, sweets, and oils/fats might be favorable for reducing the future risk of abdominal obesity, particularly in middle-aged and older Korean adults.

## 1. Introduction

Obesity is a global health issue [1]. In Korea, obesity, especially abdominal obesity, has been steadily increasing, and the prevalence of abdominal obesity has increased from 19.0% in 2009 to 23.9% in 2019 [2]. Abdominal obesity is a risk factor for metabolic syndrome and is associated with a high risk of dyslipidemia, insulin resistance, diabetes mellitus, systemic inflammation, cardiovascular disease, cancer, digestive disease, arthritis, and all-cause mortality [3,4,5]. Previous studies have explored various risk factors for abdominal obesity. Increased intake of animal foods, saturated fats, processed foods containing additives [6], and irregular meals [7] are positively associated with the risk of abdominal obesity. In contrast, the intake of fruits and vegetables [8], dairy products [9], and phytochemicals [10,11] is inversely associated with the risk of abdominal obesity.

Epidemiological studies on dietary factors are being actively conducted to identify dietary patterns associated with chronic diseases because they have emerged as a leading cause of death [12]. However, the single-nutrient approach commonly used in nutritional epidemiology studies has limitations in identifying the differences between nutrients and is unable to detect small effects of single nutrients on disease [13,14]. Thus, a dietary pattern approach that considers the interactions between different nutrients or foods has been proposed as an alternative to overcome the limitations of the conventional single-nutrient approach in understanding diet–disease relationships [15,16]. The dietary pattern approach might have significant public health implications because it allows the public to easily interpret their overall dietary intake compared to a single-nutrient or single-food approach [17,18]. In addition, nutrients are often consumed in combination, and assessing dietary patterns rather than the effects of individual nutrients may enable a more comprehensive evaluation of the overall impact of diet on disease [6].

Previous studies on dietary patterns have demonstrated an association between abdominal obesity and specific dietary patterns. In a cross-sectional study of Mexican adults aged 20–59 years, Westernized and diverse dietary patterns were positively associated with overweight, obesity, and abdominal obesity in men [19]. In addition, the “rice–vegetable” and “noodle” dietary patterns are positively associated with the likelihood of abdominal obesity among Korean women [20]. In a Japanese study of middle-aged and older men, healthy eating habits were negatively associated with waist circumference and visceral fat [21]. Another study conducted in rural China on the role of dietary patterns in abdominal obesity showed that a diet with a high intake of staple foods and red meat and a low intake of fresh fruits, vegetables, and dairy products was positively associated with visceral fat index and dyslipidemia [22].

As the prevalence of abdominal obesity continues to increase in Korea, identifying the dietary patterns associated with abdominal obesity is becoming increasingly important. Therefore, using data from the Korean Genome and Epidemiology Study (KoGES), this study aimed to identify major dietary patterns in middle-aged and older Korean populations and investigate their risk of developing abdominal obesity.

## 2. Materials and Methods

### 2.1. Data Source and Study Participants

The cause-and-effect relationship between dietary patterns and chronic diseases was examined using data from the KoGES, which was conducted to assess environmental and genetic factors as determinants of chronic diseases among the Korean population [23]. A baseline survey was conducted from 2004 to 2013 among 173,342 men and women aged 40–79 years in the Health Examinee Study (HEXA) cohort, a subset of the KoGES, who visited hospitals and health centers nationwide for medical checkups every two years. Using structured questionnaires, data regarding examinations and blood and urine tests were collected along with the survey (demographic and sociological characteristics, history of chronic diseases, family history of diseases, and meal behaviors). Abdominal obesity was the outcome variable in this study.

**Figure 1:**
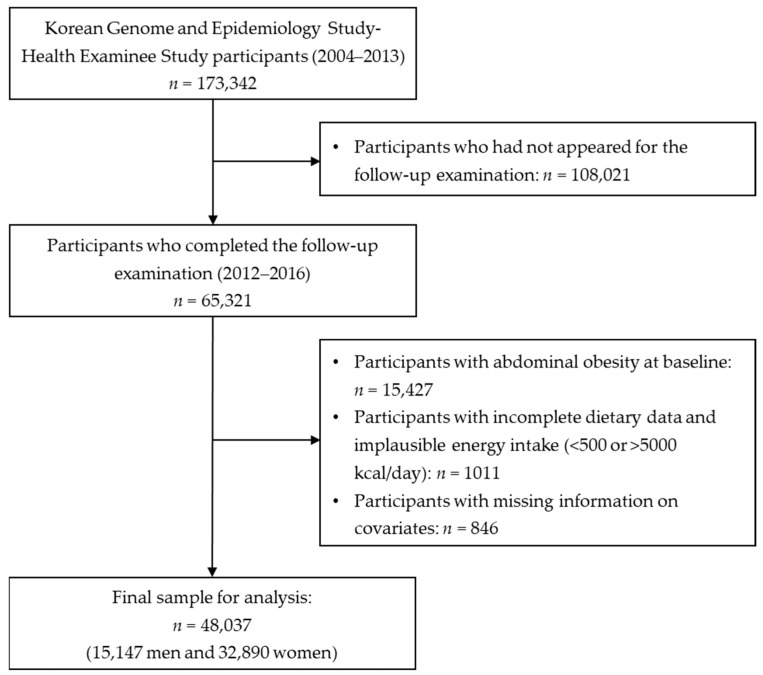
Flowchart of study population selection.

This study used data from 65,321 participants in the KoGES-HEXA cohort who underwent baseline and follow-up examinations to investigate the relationship between dietary patterns and abdominal obesity (Figure 1). We excluded participants with abdominal obesity at baseline (*n* = 15,427), no dietary data (*n* = 598), implausible energy intake (<500 kcal/day or >5000 kcal/day) (*n* = 413), and insufficient information on covariates (*n* = 846). Ultimately, 48,037 adults (15,147 men and 32,890 women) were included in the analysis of the relationship between dietary patterns and abdominal obesity. All methods and protocols were conducted in accordance with relevant institutional guidelines and regulations, and all participants provided written informed consent. This study was reviewed and approved by the Institutional Review Board (IRB) of the Korea National University of Education (IRB no. KNUE-202208-BM-0322-01).

### 2.2. Dietary Pattern Analysis

In this study, factor analysis was conducted using various food and beverage groups consumed by Korean adults in order to investigate the relationship between individuals’ general characteristics, differences in nutrient and food intake, and the incidence of abdominal obesity according to their dietary patterns.

Dietary data collected through a food frequency questionnaire (FFQ) were used to determine the dietary patterns in the KoGES-HEXA cohort. The KoGES-HEXA cohort used a semi-quantitative FFQ, which included 106 food items, to determine the usual dietary intake of the study participants [24]. Trained researchers asked the participants about the frequency of consumption of each of the listed foods and beverages over the past year and the quantity they consumed per serving. The daily nutrient intake of the participants was calculated based on their usual intake of 106 food and beverage items. The 106 food and beverage items were categorized into 24 food groups (Table 1), using the classification criteria and food group characteristics used in previous studies [25,26,27,28].

Dietary patterns were extracted through factor analysis, and separate dietary patterns were derived for men and women. Principal component analysis was performed based on the energy-adjusted total daily intake of the 24 categorized food groups using the residual method [29]. The number of factors was determined by referring to a scree plot and eigenvalues (≥1.6), and the varimax rotation method was used to increase the explanatory power of the factors. Food groups with factor loading values of ≥|0.300| were defined as significant contributors to each dietary pattern, and patterns were characterized based on those food groups.

### 2.3. Definition of Abdominal Obesity

The main outcome variable was the incidence of abdominal obesity, defined as a waist circumference ≥90 cm for men and ≥85 cm for women, according to the Korean Society for the Study of Obesity [30], during the follow-up period.

### 2.4. Statistical Analyses

Baseline sociodemographic and lifestyle characteristics across the quartiles of dietary pattern scores were examined using multivariable linear regression for continuous variables and the chi-squared test for categorical variables. Multivariable Cox proportional-hazard models were used to calculate hazard ratios (HRs) and 95% confidence intervals (CIs) to examine the relationship between dietary patterns and the incidence of abdominal obesity. Covariates included age (years, continuous), examination site, educational level (elementary school, middle school, high school, or college), smoking status (non-smoker, past smoker, or current smoker), total alcohol intake (grams, continuous), regular physical activity (yes or no), and body mass index (kg/m^2^, continuous) at the baseline examination. Linear trends across quartiles of each dietary pattern score were examined. All statistical analyses were performed using SAS software (version 9.4; SAS Institute, Cary, NC, USA).

## 3. Results

### 3.1. Factor Analysis and Dietary Patterns

Dietary patterns were identified using factor analysis according to sex. The factor-loading matrices for each dietary pattern are shown in Table 1. The three major dietary patterns accounted for 29.65% and 28.44% of the total variance in men and women, respectively. The first dietary pattern, named the “healthy” pattern, was characterized by a high consumption of white and green/yellow vegetables, fish, seaweeds, mushrooms, fruits, tubers, soy products, kimchi and pickled vegetables, milk and yogurt, eggs, nuts, and tea. The second dietary pattern was characterized by a high consumption of coffee, sweets, and oils/fats, named the “coffee and sweets” pattern. The third dietary pattern was named the “multi-grain” pattern, characterized by a very high consumption of multi-grain rice and a very low consumption of white rice, red meat/high-fat red meat, poultry, flour-based foods, processed meat/red meat by-products, and carbonated beverages.

### 3.2. General Characteristics of Study Participants with Dietary Patterns at Baseline

Study participants’ general characteristics at baseline across the quartiles of the dietary pattern scores are shown in Table 2 and Table 3. Men and women with the “healthy” pattern were more likely to have a higher educational level, be married, and exercise regularly, and were less likely to be current smokers. The “healthy” pattern was positively associated with age in men and women. Individuals with a high score for the “coffee and sweets” pattern were more likely to be younger, obese, less physically active, current smokers, or alcohol consumers. The “coffee and sweets” pattern was inversely associated with education level in men and positively associated with education level in women. The “multi-grain” pattern was identified among men and women. It was positively associated with age and regular physical activity and negatively associated with education level, current smoking, and alcohol consumption.

### 3.3. Energy and Nutrient Intake of Study Participants Based on Dietary Patterns at Baseline

The total energy and nutrient intake across the quartiles of dietary pattern scores are presented in Table 4 and Table 5. Both men and women in the highest quartiles of the “healthy” pattern had a higher intake of total energy, calcium, sodium, and dietary fiber. Furthermore, they obtained more energy from total protein (including animal and plant protein) and fat, while deriving less energy from carbohydrates compared to those in the lowest quartiles. The “coffee and sweets” pattern scores displayed an increasing trend in a percentage of energy from fat intake, accompanied by a decreasing trend in the proportion of energy from carbohydrates, total protein, animal and plant protein, and dietary fiber in both men and women. Notably, there was a sex difference in the trend in total energy intake across the “coffee and sweets” pattern scores, namely a significant positive association for men and a significant negative association for women. Meanwhile, men and women in the highest quartiles of the “multi-grain” pattern had lower intakes of total energy, total and animal protein, fat, calcium, and sodium but consumed more energy from carbohydrates and plant protein compared with those in the lowest quartiles.

### 3.4. Association of Dietary Patterns with the Incidence of Abdominal Obesity

During a mean follow-up of 4.89 years, we identified 5878 (12.24%; 1932 men and 3946 women) new cases of abdominal obesity. The association between dietary patterns and the incidence of abdominal obesity is shown in Table 6. The “healthy” pattern was negatively associated with the incidence of abdominal obesity in men and women. Individuals in the highest quartile had a lower incidence of abdominal obesity than those in the lowest quartile of the “healthy” pattern (HR for fourth vs. first quartile: 0.86, 95% CI: 0.75–0.98, *p* for trend = 0.0358 for men; HR for fourth vs. first quartile: 0.90, 95% CI: 0.83–0.99, *p* for trend = 0.0188 for women) in the fully adjusted model taking into account age, examination site, educational level, smoking status, total alcohol intake, regular physical activity, and body mass index. The “coffee and sweets” pattern showed a positive association with the incidence of abdominal obesity. Compared to individuals in the lowest quartile of the “coffee and sweets” pattern, those in the highest quartile had a higher incidence of abdominal obesity (HR for fourth vs. first quartile: 1.23, 95% CI: 1.08–1.40, *p* for trend = 0.0495 for men; HR for fourth vs. first quartile: 1.14, 95% CI: 1.04–1.25, *p* for trend = 0.0096 for women). The “multi-grain” pattern had no association with the incidence of abdominal obesity in both men and women.

## 4. Discussion

In this large-scale prospective population-based cohort study, we identified three major dietary patterns among middle-aged and older Korean adults: “healthy”, “coffee and sweets”, and “multi-grain” dietary patterns. During a mean follow-up of 4.89 years, 5878 new cases of abdominal obesity were reported, and significant associations between two of the dietary patterns and the incidence of abdominal obesity were observed in men and women. The “healthy” dietary pattern, characterized by a high consumption of white and green/yellow vegetables, fish, seaweeds, mushrooms, fruits, tubers, soy products, kimchi and pickled vegetables, milk and yogurt, eggs, nuts, and tea, was found to be protective against abdominal obesity. In contrast, the “coffee and sweets” dietary pattern increased the future risk of developing abdominal obesity, whereas the “multi-grain” dietary pattern did not exhibit any significant associations.

According to this study, women who followed a “healthy” dietary pattern consumed more vegetables, fish, seaweed, fruits, and soy products than those who did not, which aligns with previous studies. Consistent with the “healthy” pattern identified in this study, “prudent” and “healthy” patterns have been previously reported to be inversely associated with the risk of abdominal obesity in older Brazilians [31], Chinese adults [32,33], and Iranian patients with type 2 diabetes [34]. According to a recent systematic review and meta-analysis, which included 13 studies, individuals in the highest category of the “healthy/prudent” patterns had a 0.81 times lower risk of abdominal obesity compared with those in the lowest category (pooled odds ratio [OR]: 0.81, 95% CI: 0.66–0.96) [35]. Previous studies have investigated the association between abdominal obesity and various dietary indices, such as the Healthy Eating Index, Mediterranean Diet Score, and Dietary Inflammatory Index [36]. Although these dietary indices stem from different theoretical backgrounds, they all promote the consumption of fruits, vegetables, whole grains, fish, and beans over red and processed meats and saturated fats [37]. The Healthy Eating Index and the Mediterranean Diet Score have shown an inverse association with increased waist circumference and the risk of abdominal obesity [38,39]. The Dietary Inflammatory Index has been positively associated with the waist-to-hip circumference ratio [37], which is consistent with the results of this study.

Dietary components constituting the “healthy” pattern in this study, including vegetables, fish, seaweed, fruits, and soy products, play a major role in reducing waist circumference and the risk of abdominal obesity. A large-scale population-based Canadian study found a potentially beneficial role for fruit and vegetable consumption. When total fruit and vegetable consumption increased by one standard deviation, body mass index (β: −0.12 kg/m^2^, 95% CI: −0.19 to −0.05), waist circumference (β: −0.40 cm, 95% CI: −0.58 to −0.23), and percentage of body fat (β: −0.30%, 95% CI: −0.44 to −0.17) significantly decreased, and total fruit consumption showed an inverse association with general (OR for <1 servings/day vs. ≥2 servings/day: 0.87, 95% CI: 0.81–0.93; *p* for trend < 0.001) and abdominal obesity (OR for <1 servings/day vs. ≥2 servings/day: 0.88, 95% CI: 0.82–0.94, *p* for trend < 0.001) [40]. In a prospective cohort study of 2067 Korean adults, frequent consumption of fruits (>4 servings/day) led to a lower incidence of abdominal obesity compared to non-consumers in men (HR: 0.61, 95% CI: 0.47–0.78, *p* for trend < 0.0001) and women (HR: 0.74, 95% CI: 0.58–0.94, *p* for trend = 0.0125) [8]. Another Korean study of 3742 adults aged between 40–69 years reported a positive relationship between the rice–vegetable dietary pattern, characterized by a high intake of steamed rice, tofu, soy products, vegetables, and seaweed, and the odds of abdominal obesity. Individuals in the highest quintile of the rice–vegetable pattern showed 1.07 times higher odds of abdominal obesity than those in the lowest quintile (OR for fifth vs. first quintile: 1.07, 95% CI: 1.01–1.16, *p* for trend < 0.05). The differences in these results might be attributed to the fact that, unlike the “healthy” dietary pattern in this study, the “rice–vegetable” pattern in the previous study included a higher intake of steamed rice [20].

Dietary fiber and antioxidant vitamins can be obtained through a plant-rich diet. The soluble fibers of fruits, vegetables, and seaweed may reduce the risk of abdominal obesity by slowing gastric emptying and increasing postprandial glucose levels and insulin sensitivity [41,42]. Additionally, chronic inflammatory conditions can induce oxidative stress by increasing the accumulation of active oxygen in the body [43], causing general and abdominal obesity by impairing metabolic function [44,45]. Antioxidant vitamins, such as vitamins A and C, in vegetables and fruits reduce oxidative stress by controlling their reaction to active oxygen [46]. For instance, vitamin A may contribute to reducing abdominal adiposity through body fat redistribution, which moves visceral fat to the subcutaneous areas [47].

The “coffee and sweets” pattern in this study exhibited high factor loadings, mostly for coffee, sweets, and oil/fats, which is comparable to previous studies [26,27]. Since 2000, coffee shops have increased in number, coffee has gained popularity, and the consumption of coffee by Koreans has steadily increased. The average annual coffee consumption per Korean adult is 377 cups, indicating that more than one cup of coffee is consumed daily [48]. However, unlike in Europe and North America, where coffee culture originated, Koreans prefer instant coffee (an instant coffee mix containing coffee powder, sugar, and creamer) over filtered coffee [49], and this preference is particularly noticeable in older populations. According to the findings of the food source ranking of total energy, the use of instant coffee ranked 14th in the total population. However, its use ranked eighth and fifth in the population aged 50–64 and ≥65 years, respectively, indicating that the older population consumed more energy through instant coffee compared to other age groups [50]. Accordingly, the “coffee and sweets” pattern was identified as one of the major dietary patterns associated with increased abdominal obesity among middle-aged and older Korean adults.

This study showed that adherence to the “coffee and sweets” pattern increased the risk of abdominal obesity. However, previous studies on the relationship between coffee consumption and general and abdominal obesity have reported contradictory results. The Health, Alcohol, and Psychosocial factors in Eastern Europe Study documented that as the frequency of coffee consumption increased, body mass index and waist circumference decreased, and the odds of abdominal obesity reduced significantly in those drinking more than two cups per day compared with those who drank less than one cup per day (OR: 0.86, 95% CI: 0.75–0.97, *p* for trend = 0.001) [51]. In a study from the Danish part of the Monitoring Trends and Determinants in Cardiovascular Disease cohort, a significant inverse association was found between coffee consumption and lower concurrent gains in body mass index (adjusted β: −0.05 kg/m^2^, 95% CI: −0.07 to −0.02), body fat percentage (adjusted β: −0.09%, 95% CI: −0.14 to −0.04), and waist circumference (adjusted β: −0.23 cm, 95% CI: −0.34 to −0.12), irrespective of sugar and cream in the coffee [52]. A cross-sectional analysis of 17,953 Korean adults aged 19–65 years showed that frequent instant coffee consumers (≥3 times/day) had higher odds of both general (OR: 1.37, 95% CI: 1.15–1.63) and abdominal obesity (OR: 1.33, 95% CI: 1.11–1.59) than non-consumers. Additionally, the association between coffee consumption and abdominal obesity differed depending on the type of coffee consumed. A study found that, compared to non-consumers, instant coffee consumers had increased odds of general and abdominal obesity, while no significant association was observed among filtered coffee consumers [53]. Another cross-sectional study in Korea confirmed the association between black and instant coffee consumption and metabolic syndrome. Women consuming more than one serving per day of black coffee showed decreased odds of metabolic syndrome (OR: 0.63, 95% CI: 0.45–0.88, *p* for trend = 0.0089) and its components, including abdominal obesity (OR: 0.71, 95% CI: 0.54–0.93, *p* for trend = 0.0138) [54].

Although the underlying mechanisms of the association between the “coffee and sweets” pattern and abdominal obesity risk are still unclear, possible explanations have been presented. Coffee contains caffeine and polyphenols, such as phenolic and chlorogenic acids [55]. These compounds reduce insulin resistance, body weight, and fat, and produce a negative energy balance through antioxidant and anti-inflammatory functions [56,57]. Filtered or black coffee mainly contains antioxidants, whereas one serving (12 g) of instant coffee contains 5.9 g of sugar and 0.96 g of saturated fatty acids from the coffee creamer [58]. Thus, following the “coffee and sweets” pattern, which has characteristics similar to instant coffee, may increase the intake of sugar and saturated fatty acids and energy consumption, leading to abdominal obesity.

To the best of our knowledge, this is the first large-scale cohort study to prospectively identify dietary patterns associated with the risk of abdominal obesity in the Korean adult population. However, it is important to acknowledge certain limitations when interpreting the findings. First, as we studied middle-aged and older adults living in a specific area of Korea, the results may not be applicable to the entire Korean population. Second, dietary intake was assessed based on a single FFQ measurement, which could have led to an underestimation or overestimation of actual intake. Additionally, we used self-reported dietary intake information; therefore, recall errors could not be eliminated. Third, factor analysis has the limitation of being subjective; the most appropriate number of factors (dietary patterns) was derived by considering eigenvalues, scree plots, and interpretability to overcome this limitation. However, some decisions regarding factor analysis, such as food group classification and a number of factors, remain arbitrary [59].

## 5. Conclusions

Three major dietary patterns in middle-aged and older Korean adults were identified in this prospective cohort study: the “healthy,” “coffee and sweets,” and “multi-grain” patterns. The “healthy” dietary pattern had an inverse association with abdominal obesity, while the “coffee and sweets” dietary pattern had a positive association. Our findings show that adherence to a diet rich in colorful vegetables, seaweed, mushrooms, fruits, tubers, soy products, and fish, and low in coffee, sweets, and oils/fats may be beneficial in reducing the incidence of abdominal obesity, particularly in middle-aged and older Korean adults. These findings emphasize the importance of establishing dietary guidelines and nutritional education to promote healthy food choices and help reduce the risk of abdominal obesity in middle-aged and older populations. Further research is required to better understand the mechanisms underlying the association between dietary patterns and abdominal obesity.

## Figures and Tables

**Table 1:** Factor loadings for the major dietary patterns identified according to a factor analysis ^a^.

Food Group	Men			Women		
	Factor 1	Factor 2	Factor 3	Factor 1	Factor 2	Factor 3
	“Healthy”	“Coffee and Sweets”	“Multi-Grain”	“Healthy”	“Coffee and Sweets”	“Multi-Grain”
White and green/yellow vegetables	0.740 ^b^			0.733		
Fish	0.603			0.566		
Seaweed	0.571			0.557		
Mushrooms	0.562			0.561		
Fruits	0.442			0.408		
Tubers	0.413			0.433		
Soy products	0.385			0.431		
Kimchi and pickled vegetables	0.333			0.337		
Milk and yogurt	0.330			0.369		
Eggs	0.313			0.353		
Nuts	0.306			0.333		
Tea	0.301			0.324		
Sweets		0.897			0.871	
Oils and fats		0.892			0.871	
Coffee		0.850			0.785	
Multi-grain rice			0.934			0.896
White rice			−0.745			−0.707
Red meat and high-fat red meat			−0.401			−0.427
Poultry			−0.359			−0.324
Flour-based foods			−0.335			−0.402
Processed meats and red meat by-products			−0.309			−0.351
Carbonated beverages			−0.309			−0.302
Dairy products						
Other beverages						
Variance of intake explained (%)	11.04	9.88	8.73	10.67	9.15	8.62
Cumulative variance of intake explained (%)	11.04	20.92	29.65	10.67	19.82	28.44

^a^ Factor analysis was performed for 24 food groups. ^b^ Factor loading values <|0.300| are not listed in the table for simplicity.

**Table 2:** General characteristics of study participants at baseline across the quartiles of each dietary pattern score in middle-aged Korean men in the KoGES-HEXA cohort.

	Quartile (Q) of Dietary Pattern Score				*p* Value ^b^
	Q1 (Lowest)	Q2	Q3	Q4 (Highest)	
“Healthy” pattern					
Age, yrs	53.9 ± 8.6 ^a^	54.4 ± 8.5	55.1 ± 8.3	56.1 ± 8.4	<0.0001
Education, college or higher, %	39.7	42.5	46.1	50.5	<0.0001
Marital status, single, %	7.8	6.1	4.3	4.7	<0.0001
Current smokers, %	30.0	27.8	26.2	22.7	<0.0001
Alcohol, g/d	15.7 ± 27.4	15.4 ± 26.5	15.6 ± 25.4	15.2 ± 27.0	0.8442
Regular physical activity, %	51.4	59.5	63.4	68.3	<0.0001
Body mass index, kg/m^2^	23.3 ± 2.3	23.4 ± 2.2	23.4 ± 2.3	23.5 ± 2.2	0.0037
“Coffee and sweets” pattern					
Age, yrs	55.4 ± 8.4	54.7 ± 8.5	55.2 ± 8.5	54.3 ± 8.5	<0.0001
Education, college or higher, %	25.6	28.2	24.9	21.4	<0.0001
Marital status, single, %	7.0	5.7	4.4	5.8	<0.0001
Current smokers, %	15.6	22.8	27.8	41.6	<0.0001
Alcohol, g/d	15.4 ± 28.1	15.3 ± 24.4	15.3 ± 25.9	16.0 ± 27.8	0.6426
Regular physical activity, %	64.9	64.7	59.2	53.8	<0.0001
Body mass index, kg/m^2^	23.2 ± 2.3	23.5 ± 2.3	23.5 ± 2.2	23.5 ± 2.3	<0.0001
“Multi-grain” pattern					
Age, yrs	52.6 ± 8.6	53.8 ± 8.6	55.4 ± 8.2	57.7 ± 7.7	<0.0001
Education, college or higher, %	44.8	50.7	46.1	37.2	<0.0001
Marital status, single, %	9.0	5.5	3.9	4.4	<0.0001
Current smokers, %	33.7	29.7	24.4	20.1	<0.0001
Alcohol, g/d	19.0 ± 32.1	17.4 ± 28.0	14.4 ± 22.4	11.2 ± 21.8	<0.0001
Regular physical activity, %	51.6	63.0	65.5	62.5	<0.0001
Body mass index, kg/m^2^	23.4 ± 2.3	23.6 ± 2.3	23.5 ± 2.2	23.2 ± 2.2	<0.0001

KoGES, Korean Genome and Epidemiology Study; HEXA, Health Examinee. ^a^ Mean ± standard deviation (all such values). ^b^ *p* values were obtained from multiple linear regression for continuous variables and the Mantel–Haenszel chi-squared test for categorical variables.

**Table 3:** General characteristics of study participants at baseline across the quartiles of each dietary pattern score in middle-aged Korean women in the KoGES-HEXA cohort.

	Quartile (Q) of Dietary Pattern Score				*p* Value ^b^
	Q1 (Lowest)	Q2	Q3	Q4 (Highest)	
“Healthy” pattern					
Age, yrs	51.6 ± 7.8 ^a^	51.9 ± 7.7	52.4 ± 7.3	52.7 ± 7.2	<0.0001
Education, college or higher, %	23.3	26.2	28.6	28.7	<0.0001
Marital status, single, %	13.5	11.6	11.2	10.8	<0.0001
Current smokers, %	1.9	1.8	1.5	1.4	0.0072
Alcohol, g/d	1.7 ± 5.9	1.6 ± 6.9	1.7 ± 6.7	1.5 ± 5.7	0.3172
Regular physical activity, %	46.6	52.7	57.9	63.9	<0.0001
Body mass index, kg/m^2^	22.8 ± 2.4	22.8 ± 2.4	22.7 ± 2.4	22.8 ± 2.4	0.0188
“Coffee and sweets” pattern					
Age, yrs	53.3 ± 7.5	52.7 ± 7.6	51.3 ± 7.3	51.4 ± 7.4	<0.0001
Education, college or higher, %	23.8	27.2	29.4	26.4	<0.0001
Marital status, single, %	12.3	12.0	10.3	12.3	0.2907
Current smokers, %	0.9	1.1	1.5	2.9	<0.0001
Alcohol, g/d	1.2 ± 5.5	1.4 ± 5.2	1.9 ± 6.4	2.0 ± 7.8	<0.0001
Regular physical activity, %	58.3	58.2	54.9	49.6	<0.0001
Body mass index, kg/m^2^	22.6 ± 2.4	22.8 ± 2.4	22.9 ± 2.4	22.9 ± 2.4	<0.0001
“Multi-grain” pattern					
Age, yrs	50.3 ± 7.4	51.1 ± 7.3	52.5 ± 7.3	54.7 ± 7.3	<0.0001
Education, college or higher, %	30.3	32.9	26.1	17.5	<0.0001
Marital status, single, %	12.5	11.1	10.6	12.8	0.8064
Current smokers, %	2.8	1.4	1.2	1.1	<0.0001
Alcohol, g/d	2.4 ± 8.0	1.9 ± 5.9	1.2 ± 4.2	1.0 ± 6.5	<0.0001
Regular physical activity, %	47.2	58.4	59.1	56.3	<0.0001
Body mass index, kg/m^2^	22.8 ± 2.5	22.7 ± 2.4	22.8 ± 2.4	22.9 ± 2.3	0.0507

KoGES, Korean Genome and Epidemiology Study; HEXA, Health Examinee. ^a^ Mean ± standard deviation (all such values). ^b^ *p* values were obtained from multiple linear regression for continuous variables and the Mantel–Haenszel chi-squared test for categorical variables.

**Table 4:** Energy and nutrient intake of study participants at baseline across the quartiles of each dietary pattern score in middle-aged Korean men in the KoGES-HEXA cohort.

	Quartile (Q) of Dietary Pattern Score				*p* Value ^b^
	Q1 (Lowest)	Q2	Q3	Q4 (Highest)	
“Healthy” pattern					
Energy, kcal/day	1717 ± 464 ^a^	1807 ± 443	1884 ± 484	1829 ± 609	<0.0001
%energy from carbohydrates	76.0 ± 5.4	73.4 ± 5.7	71.8 ± 6.3	68.9 ± 8.1	<0.0001
%energy from total protein	10.6 ± 1.5	11.8 ± 1.6	12.9 ± 1.9	14.8 ± 2.9	<0.0001
%energy from animal protein	3.3 ± 1.7	4.5 ± 2.0	5.4 ± 2.2	7.0 ± 3.2	<0.0001
%energy from plant protein	7.3 ± 0.8	7.3 ± 0.9	7.4 ± 1.0	7.8 ± 1.3	<0.0001
%energy from fat	10.8 ± 4.5	12.8 ± 4.6	13.8 ± 4.8	15.8 ± 6.0	<0.0001
Calcium, mg/day	306.3 ± 160.1	410.1 ± 174.6	488.0 ± 202.4	602.5 ± 273.4	<0.0001
Sodium, mg/day	992 ± 568	1297 ± 653	1588 ± 838	2026 ± 1168	<0.0001
Dietary fiber, g/day	7.4 ± 4.0	10.1 ± 4.2	12.7 ± 5.3	16.3 ± 7.7	<0.0001
“Coffee and sweets” pattern					
Energy, kcal/day	1749 ± 489	1841 ± 554	1866 ± 460	1780 ± 514	<0.0001
%energy from carbohydrates	73.5 ± 7.1	72.1 ± 7.2	72.5 ± 6.3	72.0 ± 7.2	<0.0001
%energy from total protein	12.6 ± 2.6	12.9 ± 2.7	12.4 ± 2.3	12.1 ± 2.6	<0.0001
%energy from animal protein	4.9 ± 2.8	5.3 ± 2.8	5.0 ± 2.4	4.9 ± 2.7	<0.0001
%energy from plant protein	7.6 ± 1.1	7.6 ± 1.1	7.4 ± 0.9	7.2 ± 1.0	<0.0001
%energy from fat	12.1 ± 5.3	13.3 ± 5.4	13.4 ± 4.7	14.3 ± 5.6	<0.0001
Calcium, mg/day	421.3 ± 249.0	465.5 ± 249.6	468.6 ± 220.4	451.5 ± 211.1	<0.0001
Sodium, mg/day	1363 ± 888	1574 ± 1006	1509 ± 876	1457 ± 898	<0.0001
Dietary fiber, g/day	11.1 ± 6.6	12.4 ± 6.9	11.9 ± 6.2	11.0 ± 6.0	<0.0001
“Multi-grain” pattern					
Energy, kcal/day	1769 ± 573	1995 ± 598	1832 ± 398	1640 ± 348	<0.0001
%energy from carbohydrates	70.4 ± 8.7	68.3 ± 6.1	73.2 ± 3.9	78.2 ± 3.4	<0.0001
%energy from total protein	12.9 ± 3.2	13.9 ± 2.5	12.4 ± 1.7	10.8 ± 1.4	<0.0001
%energy from animal protein	5.8 ± 3.4	6.6 ± 2.6	4.8 ± 1.6	3.0 ± 1.3	<0.0001
%energy from plant protein	7.1 ± 1.0	7.4 ± 1.1	7.6 ± 1.0	7.8 ± 0.9	<0.0001
%energy from fat	14.9 ± 6.8	16.6 ± 4.4	12.8 ± 2.9	8.8 ± 2.6	<0.0001
Calcium, mg/day	426.6 ± 223.3	564.1 ± 264.8	469.0 ± 215.7	347.3 ± 166.9	<0.0001
Sodium, mg/day	1459 ± 895	1858 ± 1059	1480 ± 841	1107 ± 696	<0.0001
Dietary fiber, g/day	9.6 ± 5.8	14.7 ± 7.2	12.5 ± 6.1	9.7 ± 4.9	<0.0001

KoGES, Korean Genome and Epidemiology Study; HEXA, Health Examinee. ^a^ Mean ± standard deviation (all such values). ^b^ *p*-values were obtained from multiple linear regressions.

**Table 5:** Energy and nutrient intake of study participants at baseline across the quartiles of each dietary pattern score in middle-aged Korean women in the KoGES-HEXA cohort.

	Quartile (Q) of Dietary Pattern Score				*p* Value ^b^
	Q1 (Lowest)	Q2	Q3	Q4 (Highest)	
“Healthy” pattern					
Energy, kcal/day	1612 ± 469 ^a^	1702 ± 459	1723 ± 518	1650 ± 627	<0.0001
%energy from carbohydrates	76.5 ± 5.8	74.2 ± 6.1	72.6 ± 6.4	69.5 ± 8.3	<0.0001
%energy from total protein	10.8 ± 1.6	12.1 ± 1.8	13.1 ± 2.0	15.1 ± 3.1	<0.0001
%energy from animal protein	3.4 ± 1.8	4.7 ± 2.1	5.6 ± 2.3	7.2 ± 3.3	<0.0001
%energy from plant protein	7.3 ± 0.9	7.4 ± 0.9	7.5 ± 1.1	7.9 ± 1.5	<0.0001
%energy from fat	10.5 ± 4.8	12.3 ± 4.8	13.5 ± 4.9	15.6 ± 5.9	<0.0001
Calcium, mg/day	318.4 ± 172.2	431.3 ± 187.7	519.3 ± 227.9	647.0 ± 337.9	<0.0001
Sodium, mg/day	912 ± 562	1211 ± 610	1474 ± 795	1868 ± 1127	<0.0001
Dietary fiber, g/day	8.2 ± 4.6	11.2 ± 4.9	13.8 ± 6.1	17.6 ± 9.6	<0.0001
“Coffee and sweets” pattern					
Energy, kcal/day	1707 ± 526	1630 ± 547	1763 ± 538	1588 ± 466	<0.0001
%energy from carbohydrates	74.0 ± 7.4	73.6 ± 7.3	72.4 ± 6.9	72.7 ± 6.9	<0.0001
%energy from total protein	12.8 ± 2.9	12.9 ± 2.7	12.9 ± 2.6	12.4 ± 2.6	<0.0001
%energy from animal protein	5.2 ± 3.0	5.2 ± 2.8	5.4 ± 2.7	5.1 ± 2.7	<0.0001
%energy from plant protein	7.6 ± 1.2	7.8 ± 1.2	7.5 ± 1.1	7.3 ± 1.0	<0.0001
%energy from fat	12.0 ± 5.5	12.4 ± 5.5	13.6 ± 5.2	13.9 ± 5.4	<0.0001
Calcium, mg/day	473.9 ± 296.6	473.6 ± 275.0	516.5 ± 275.5	452.0 ± 217.4	<0.0001
Sodium, mg/day	1322 ± 866	1401 ± 957	1470 ± 898	1271 ± 767	<0.0001
Dietary fiber, g/day	13.0 ± 8.0	12.9 ± 7.9	13.5 ± 7.6	11.3 ± 5.9	<0.0001
“Multi-grain” pattern					
Energy, kcal/day	1678 ± 639	1761 ± 585	1692 ± 457	1556 ± 346	<0.0001
%energy from carbohydrates	69.2 ± 9.2	70.1 ± 5.3	74.5 ± 4.3	78.9 ± 3.7	<0.0001
%energy from total protein	13.7 ± 3.4	13.7 ± 2.4	12.5 ± 2.0	11.1 ± 1.7	<0.0001
%energy from animal protein	6.5 ± 3.6	6.3 ± 2.4	4.9 ± 1.8	3.2 ± 1.5	<0.0001
%energy from plant protein	7.2 ± 1.2	7.4 ± 1.1	7.6 ± 1.0	7.9 ± 1.0	<0.0001
%energy from fat	16.1 ± 7.1	15.5 ± 3.7	12.0 ± 3.0	8.3 ± 2.7	<0.0001
Calcium, mg/day	491.0 ± 282.7	549.0 ± 289.4	491.8 ± 257.1	384.2 ± 212.1	<0.0001
Sodium, mg/day	1468 ± 938	1526 ± 891	1344 ± 830	1126 ± 793	<0.0001
Dietary fiber, g/day	12.1 ± 7.5	14.6 ± 7.9	13.3 ± 7.4	10.8 ± 6.4	<0.0001

KoGES, Korean Genome and Epidemiology Study; HEXA, Health Examinee. ^a^ Mean ± standard deviation (all such values). ^b^ *p*-values were obtained from multiple linear regressions.

**Table 6:** Adjusted hazard ratios (95% confidence intervals) of abdominal obesity across the quartiles of each dietary pattern score in middle-aged Korean adults in the KoGES-HEXA cohort.

	Quartile (Q) of Dietary Pattern Score				*p* for Trend ^b^
	Q1 (Lowest)	Q2	Q3	Q4 (Highest)	
Men					
“Healthy” pattern					
Person years	19,070	18,308	17,803	18,166	-
Abdominal obesity (cases)	486	487	480	479	-
Rate per 1000 person years	25.5	26.6	27.0	26.4	-
Age-adjusted HR (95% CI) ^a^	1.00	1.10 (0.97–1.25)	1.20 (1.06–1.36)	1.10 (0.97–1.25)	0.1286
Multivariate-adjusted HR (95% CI) ^b^	1.00	0.90 (0.80–1.03)	1.00 (0.88–1.14)	0.86 (0.75–0.98)	0.0358
“Coffee and sweets” pattern					
Person years	18,641	18,494	18,209	18,003	-
Abdominal obesity (cases)	413	500	510	509	-
Rate per 1000 person years	22.2	27.0	28.0	28.3	-
Age-adjusted HR (95% CI)	1.00	1.24 (1.09–1.41)	1.32 (1.16–1.50)	1.35 (1.18–1.53)	<0.0001
Multivariate-adjusted HR (95% CI)	1.00	1.10 (0.97–1.26)	1.12 (0.98–1.28)	1.23 (1.08–1.40)	0.0495
“Multi-grain” pattern					
Person years	18,731	18,242	18,146	18,228	-
Abdominal obesity (cases)	495	507	480	450	-
Rate per 1000 person years	26.4	27.8	26.5	24.7	-
Age-adjusted HR (95% CI)	1.00	1.09 (0.96–1.23)	1.04 (0.92–1.18)	0.95 (0.84–1.09)	0.7467
Multivariate-adjusted HR (95% CI)	1.00	0.94 (0.78–1.14)	0.98 (0.78–1.22)	1.05 (0.82–1.34)	0.6128
Women					
“Healthy” pattern					
Person years	40,175	39,901	39,904	41,522	-
Abdominal obesity (cases)	1013	940	990	1003	-
Rate per 1000 person years	25.2	23.6	24.8	24.2	-
Age-adjusted HR (95% CI)	1.00	0.94 (0.86–1.03)	0.99 (0.90–1.08)	0.88 (0.81–0.96)	0.0111
Multivariate-adjusted HR (95% CI)	1.00	0.92 (0.84–1.01)	0.91 (0.83–0.99)	0.90 (0.83–0.99)	0.0188
“Coffee and sweets” pattern					
Person years	41,011	40,451	40,296	39,744	-
Abdominal obesity (cases)	832	984	1051	1079	-
Rate per 1000 person years	20.3	24.3	26.1	27.1	-
Age-adjusted HR (95% CI)	1.00	1.25 (1.14–1.37)	1.49 (1.36–1.63)	1.50 (1.37–1.64)	<0.0001
Multivariate-adjusted HR (95% CI)	1.00	1.06 (0.97–1.16)	1.11 (1.01–1.22)	1.14 (1.04–1.25)	0.0096
“Multi-grain” pattern					
Person years	31,203	39,838	40,046	40,415	-
Abdominal obesity (cases)	958	961	954	1073	-
Rate per 1000 person years	30.7	24.1	23.8	26.5	-
Age-adjusted HR (95% CI)	1.00	1.07 (0.98–1.17)	0.99 (0.91–1.09)	0.99 (0.90–1.08)	0.7757
Multivariate-adjusted HR (95% CI)	1.00	0.99 (0.89–1.11)	0.97 (0.86–1.10)	0.97 (0.86–1.10)	0.4981

KoGES, Korean Genome and Epidemiology Study; HEXA, Health Examinee; Q, quartile; HR, hazard ratio; CI, confidence interval. ^a^ The age-adjusted model was adjusted for age (years, continuous); the multivariate-adjusted model was adjusted for age (years, continuous), examination site, educational level (elementary school, middle school, high school, or college), smoking status (non-smoker, past smoker, or current smoker), total alcohol intake (grams, continuous), regular physical activity (yes or no), and body mass index (kg/m^2^, continuous). ^b^ *p* for the trend was calculated using the median value of each dietary pattern score in each quartile, treating it as a continuous variable in the model.

## Data Availability

The data presented in this study are available on request from the corresponding authors upon reasonable request. The data are not publicly available due to privacy protection of people who participated in the KoGES-HEXA cohort.
